# How Opioids Changed What We Thought About Addiction and the Implications for Public Health: An Editorial

**DOI:** 10.7759/cureus.40181

**Published:** 2023-06-09

**Authors:** Joseph Pergolizzi, Jo Ann K LeQuang

**Affiliations:** 1 Clinical Research and Healthcare Policy, Nema Research, Naples, USA; 2 Healthcare Policy, Nema Research, Naples, USA

**Keywords:** disease model of addiction, addiction, opioid addiction, public health, opioid use disorder

## Abstract

Dealing with substance use disorder is moving out of the realm of criminality, morality, and law enforcement toward a more medically grounded approach. This was particularly evident when opioid use disorder, which started roughly around 1999 and has continued to increase over the decades, was observed to affect mainly White people. This has driven a re-assessment of the nature of addiction. The previous big drug epidemic involved crack cocaine which was criminalized to the point that many users drew harsh prison sentences. Crack addiction was seen as a crime. Of course, crack was a drug predominantly used by Black people. The emergence of a White drug addict prompted a re-evaluation of what addiction meant and how it might be treated. This has led to neuropsychiatric evaluations of substance use disorder and the notion that opioid use disorder is a disease rather than a moral failure. Treating opioid use disorder as a physiological disorder caused by prolonged exposure to a drug that has the ability to rewire a healthy brain to drive it to compulsively seek more drugs appears to be a reasonable, compassionate, and scientifically sound approach to substance use disorder. This may lead to effective ways to treat or manage opioid use disorder. While this is a good thing, it is regrettable that such measures were not considered when the drug epidemic affected racial or ethnic minorities with less political influence and social clout. In other words, seeing opioid use disorder as, first and foremost, a disease rather than a crime is enlightened, even if we did not take the most enlightened path to get there.

## Editorial

Addiction has long existed, but addiction among a predominantly White population, including those with health insurance and high socioeconomic status, was something novel. When addiction occurred in small, marginalized, and disenfranchised communities, it was easy to dismiss it as a moral failing and a criminal act. Earlier cycles of crack cocaine addiction affected mainly Black Americans [1, 2]. The idea that compulsive opioid use affected mainly White people challenged public health assumptions about the social determinants of drug use, in that this new group of white people with opioid use disorder often had moderate to high socioeconomic status, good education, and health insurance. These were largely the people of privilege. Seeing them struggle with compulsive opioid cravings and dope-sickness brought certain misconceptions to light.

Addiction in White people was studied seriously, revealing the underlying neuropsychiatric underpinnings of addiction. This neurological component suggested that opioids rewired parts of the brain, particularly the reward circuits, to the point that addiction had to be regarded as a brain disease. With that, the word "addiction" became relegated to the linguistic scrapheap as stigmatizing, replaced by "opioid use disorder," which the Centers for Disease Control and Prevention (CDC) defined as a "treatable, chronic disease" adding that it can affect anyone regardless of age, race, gender, income level, or class status [3]. Stigmatization of drug users was not even a consideration when those abusing the drugs were people of color, but care was taken to make sure White people were not unduly branded with words that carry negative connotations. Opioid use disorder was taken up as a condition better handled by psychiatrists than law enforcement, although the American Psychiatric Association stopped short of the disease language, calling it a disorder [4]. Indeed, the distinction between a disorder and a disease is contentious and possibly unimportant from a medical standpoint, but it quite effectively shifts the discussion away from morality and criminality toward physiology and medical science. Addiction emerged as a brain disease; there may even be a genetic component involved.

This was a jaw-dropping about-face for a society that had a few decades earlier had ruthlessly criminalized the use of crack cocaine. From the 1980s to the turn of the millennium, the "war on drugs" resulted in numerous incarcerations for crack cocaine, which disproportionately affected the Black population. Crack was a "Black drug of choice," and then-President Bill Clinton allowed for harsher penalties to be applied to crack than to "soft" or powder cocaine, which was essentially the same drug, just the form more apt to be used by wealthy White people. When the appeal of crack waned, opioids emerged as the main drug of abuse in the nation; since opioids were favored by White people, politicians, lawmakers, and the healthcare establishment had to reconsider whether drug use was really criminal [5].

With this transition, neurotoxicity and neuroplasticity became buzzwords in addiction medicine (one of the few areas where the word "addiction" is still tolerated). This allowed the old notion of drug abuse to transition away from morality and legality toward a more medically understood condition. This is an important course correction in our ongoing efforts against substance use disorder, particularly as we apply it to all substances of abuse.

In the meantime, the opioid epidemic itself has transitioned, going from diverted pharmaceutical products to synthetic opioids, such as illicit fentanyl cooked up in clandestine labs. Today, opioid use disorder is prevalent in all racial and ethnic communities, with White people still in the majority. Opioid use disorder remains criminalized and continues to be misunderstood. While the disease model of opioid use disorder is gaining traction, it is not as easy a fit as one might assume.

For example, if opioid use disorder is a terrible disease, it stands to reason that people with this disease would want to overcome it, but this sentiment is not shared by many of those with opioid use disorder. After all, who among us with a brain tumor would not want it removed? Using the disease model, it is hard to imagine a person with cancer refusing to give up cancer, but such paradoxes exist in the community of those with opioid use disorder. In fact, part of the compulsive nature of opioid use disorder is a desire to keep taking opioids, even when the user wants to stop and fully understands that continuing to use opioids is not beneficial. It is this reticence to rehabilitate from opioid use disorder, even when options for rehabilitation are available, that makes it hard for people to truly sympathize with those in the throes of opioid use disorder. Yet this compulsive aspect may be inherent in the perverse nature of the disease. Prolonged exposure to opioids can result in physiologic dependence, such that abrupt discontinuation can result in withdrawal symptoms. Unlike other diseases, which can often be stabilized and managed long-term with specific treatments, opioid use disorder is sometimes viewed as a zero-sum gain of total abstinence or total surrender to the drug. Medication-assisted treatment may be a middle route; like insulin to a diabetic, buprenorphine or methadone maintenance may help a person with the disease to maintain a productive and stable life. Seeing that opioid use disorder has a psychiatric as well as physiologic aspect is also a step forward, even if effective psychiatric interventions for opioid use disorder are still evolving. Innovative new therapies, drugs, and devices are bringing new hope. But why didn't we start to search for effective treatments for substance use disorder when addiction was something that happened to poor minorities?

 Although we are starting to view opioid use disorder and other forms of substance abuse as physiologic manifestations of prolonged exposure to neurotoxic agents, the ugly side of addiction make it hard to break through to a purely medical model of opioid use disorder. But if we want to cling to the old paradigm of drug use as a moral flaw and a crime, we have to admit it has not in any way helped us to alleviate opioid use in the United States. With this medical model of opioid use disorder, we now have a brave new model of drug use disorder and a hesitation to use it. This may be due to the fact that it is a radical about-face. It may be caused by the fact that there are criminal aspects to drug use, particularly in drug importation and sales. It may also derive from the fact that people who deal for prolonged periods with those who suffer from opioid use disorder often experience compassion burnout and fatigue. There is much that is ugly about opioid use disorder, and we would be wise to admit it.

Had opioid addiction remained in the world of street heroin or illicit fentanyl cooked up in clandestine labs and had those taking these drugs been poor people of color with limited influence in society and marginalized clout, we might all still view opioid use disorder as a moral failing and a crime worthy of prolonged incarceration. While it is important that we have emerged from our old view of addiction as criminality, it points to the determinants of health that we only started to dig deeply into the real mechanisms of addiction once it affected white people. In this case, studying addiction as a medical or physiological phenomenon was an important and enlightened step. It just should have happened sooner and with other drugs in other communities.

This does not detract from the fact that we have learned a lot from the fact that opioids were so widely used among white people. It appears to have compelled a less emotional and more scientific exploration of opioid use. It prompted explorations that went beyond a moralistic assessment of opioid use and considered physiologic explanations. It also allowed us to assess the role of criminalization of drug use and how poorly that model has served the public good. It inspired discoveries for ways to manage this condition in healthful and humane ways.

Of course, enlightenment comes slowly with many speed bumps. Opioid use is still a criminal offense in many parts of the country. Those who know people with opioid use disorder know the frustration of dealing with difficult and seemingly illogical people. It is nearly impossible to observe the downfall of major cities, homeless encampments, and used syringes on urban streets and not feel a sense of revulsion and anger. How did we fall so far so fast?

It is clear that regarding opioid use disorder as a free choice by independent agents is wrong. Further, it is wrong to think that those with rewired reward circuits in their brains can simply walk away from drugs. But it must also be clear that opioid use disorder is not something we can ignore. We just must pay attention to it in the right ways.

We have entered a neuropsychiatric age of discovery in terms of understanding neurotransmission and how various substances can alter brain circuitry. We understand the potential comorbidities and associations, such as mental health disorders, that can drive addiction. But we must also move confidently into this new era with a more enlightened philosophy, namely that when we are challenged by medical puzzles, such as addiction, we investigate them without regard to the populations they affect. We must not allow race, ethnicity, national origin, religion, or other demographic drivers or social determinants to influence how much care and science we invest in solving a problem. We cannot view addiction as a crime in one population and a disease in another. And we must recognize that opioid use disorder - whether or not we count it as a disease - is a condition that is going to challenge our medical minds and our professional compassion.
